# Long COVID and Unemployment in Hawaii

**DOI:** 10.3390/ijerph20136231

**Published:** 2023-06-27

**Authors:** Carl Bonham, Ruben Juarez, Nicole Siegal

**Affiliations:** Department of Economics and UHERO, University of Hawaii, 2424 Maile Way, Saunders 542, Honolulu, HI 96822, USA; bonham@hawaii.edu (C.B.); nsiegal@hawaii.edu (N.S.)

**Keywords:** COVID-19, long COVID, employment

## Abstract

The state of Hawaii has seen 390,000 COVID-19 cases and nearly 1900 deaths since the start of the pandemic. Although the negative impact of the pandemic on employment has been widely documented, this paper demonstrates that those who were infected and suffer from lingering symptoms (i.e., long COVID) had different employment outcomes than those who did not experience such symptoms. Using data from our longitudinal cohort in the state of Hawaii, we found that those who reported long COVID in May 2022 were 6.43% more likely to be unemployed at the time of the May survey and 7.07% more likely in November 2022. In addition, we showed that vaccination is associated with higher rates of employment; each additional vaccine an individual received by May decreased the likelihood of unemployment by 6.9% in May and 3.9% in November. Further, individuals who reported more severe symptoms of long COVID were 6.36% less likely to be employed in May and 5.75% less likely to be employed in November. Our results suggest that vaccination policies and policies aimed at preventing contraction and accommodating individuals with long COVID may be effective measures for mitigating the adverse effects of the pandemic on employment.

## 1. Introduction

From March 2020 to May 2023, almost 390,000 cases and 1900 fatalities were reported in Hawaii, according to the Department of Health [1]. Studies suggest that lingering symptoms, commonly known as long COVID, occurred in 8–30% of infections [2,3]. Prolonged symptoms can have significant medical implications for physical and cognitive health, affecting daily life by causing mental fog, persistent coughing, and other conditions [4]. In addition to physical and cognitive health, long COVID may have social and economic impacts, which have yet to be measured.

Most of the literature evaluating social and economic impacts has focused on the overall impact of COVID and previous pandemics. Researchers evaluated similar historical outbreaks, such as the Black Death and 1918 influenza, to understand how future economic and societal trends may mimic the past. It has been found that large-scale death and disease move populations to lower growth paths in terms of population and urbanization [5], employment [6], and economic growth [7]. In terms of the effect of pandemics on per capita income, the evidence is mixed. Large-scale death can lead to labor shortages, which may drive up wages. For instance, during the 1920s, each additional death per thousand from the 1918 influenza resulted in an average annual increase of at least 0.15 percent in the rate of growth of real per capita income over the following decade [8]. However, in non-Malthusian economies in earlier periods, incomes were observed to decrease [9]. Disease outbreaks have been associated with increases in both income and wealth inequality; however, they are noted to lead to health inequalities when lower socioeconomic populations have a higher prevalence of contraction, which can lead to ongoing health complications or death [10].

Focusing specifically on the economic impacts evaluated thus far for the COVID pandemic in the United States, the costs to employment, growth, and government spending have spurred numerous studies. The costs related to increased healthcare usage and productivity losses due to infection alone were estimated to cost USD 163.4 billion [11]. Additional preliminary impacts suggest COVID-19 illnesses reduced the labor force by approximately 500,000 people (0.2% of adults), with an estimated USD 9000 in forgone earnings per COVID-19 absence from work [12]. Furthermore, geographic disparities in terms of economic losses were observed. Specifically, areas that were affected earlier by the pandemic experienced higher fatality rates among infected individuals and more significant economic losses. However, it is important to note that areas with reduced mobility did not exhibit significantly different economic outcomes compared to other regions [13]. Macroeconomic modeling estimates suggest that the supply shocks to the economy during the pandemic led to an overreaction in demand, resulting in a demand-deficient recession [14]. Notably, while the state of Hawaii had lower cases and mortality rates than much of the country, its economy experienced a slower recovery due in part to its dependence on tourism, which began to trend upward after other industries [13]. As of April 2022, non-farm employment in Hawaii remained 10% lower than pre-pandemic levels, with many workers not returning to the labor force or leaving the state [15].

Another strand of the literature showed the considerable health impacts of long COVID on individuals’ mental and physical capacities. Common ongoing symptoms include fatigue, fever, respiratory difficulty, chest pains, heart palpitations, brain fog, headaches, digestive distress, and joint or muscle pain, among others. In the most severe cases, long COVID can lead to damage to organ systems and mental health impairment [2]. Treatment for these conditions varied depending on symptoms and severity, with the estimated annual cost of long COVID treatment being between USD 43 billion and 172 billion, and the estimated total lost income to sufferers being between USD 101 billion and 430 billion [16].

Although the impact of long COVID on the physical and mental health of individuals has been widely documented, there is limited evidence that long COVID has ongoing effects on aspects of life, such as long-term employment. Evidence from this has been provided by limited studies, including a survey of 15,000 adults in the U.S., which found that individuals with long COVID had a roughly 4% higher unemployment rate than those without lingering symptoms [17]. Based on long COVID rates and employment patterns, a 2022 Brookings report estimated that long COVID could have accounted for 15% of the nation’s 10.6 million unfilled jobs in January 2022 [18]. Other work, using New York state insurance claims, found that 40% of workers with long COVID returned to work within 60 days of infection while still undergoing treatment, while 18% could not return to work for more than one year [19]. In mid-2020, Swedish workers with long COVID took more and prolonged sick leave than other sick workers during the pandemic’s initial phase [20].

Building on this literature, we have three hypotheses: (1) We hypothesize that long COVID has a negative effect on employment outcomes. Individuals experiencing long COVID may face challenges in maintaining employment or finding new job opportunities due to persistent symptoms and health complications. (2) We hypothesize that vaccination has a positive effect on employment outcomes. Vaccination against COVID-19 may reduce the risk of developing long COVID or experiencing severe symptoms, thus improving individuals’ ability to participate in the labor market. (3) We hypothesize that the severity of long COVID symptoms is associated with an increased likelihood of unemployment. Individuals with more severe symptoms may face additional difficulties in performing job tasks, leading to job loss or reduced employment opportunities.

To investigate these hypotheses, our study is the first to examine the relationship between long COVID and unemployment in the context of Hawaii. To be specific, we examine whether there are significant differences in employment status between individuals suffering from long COVID and those with no lingering symptoms. Additionally, we examine whether these differences persisted over time and how symptom severity, duration, and vaccination status affected employment outcomes. Unlike the existing literature, we carefully control for the number of COVID-19 vaccine shots, the length and severity of symptoms, and various demographic variables (age, sex, race, and education). In particular, from our longitudinal cohort of participants, we found that individuals who reported long COVID in May 2022 were 6.4% more likely to be unemployed at the time of the survey and 7.1% more likely in November 2022. Furthermore, our results indicate that the likelihood of unemployment increased with the severity of symptoms experienced by individuals. Individuals who reported more severe symptoms of long COVID were found to be 6.36% less likely to be employed in May and 5.75% less likely to be employed in November. In addition, we showed that vaccination is associated with higher employment rates, with each additional vaccine shot an individual received by May decreasing the likelihood of unemployment by 6.9%.

This study has significant implications for public policy and public health, aligning with a significant scope of the *International Journal of Environmental Research and Public Health*. Understanding the impact of long COVID on employment is crucial for developing targeted strategies and support systems to mitigate the negative effects on individuals’ livelihoods and the overall labor market. Our findings emphasize the pressing need for policies that target the prevention of COVID-19 infections and address the needs of individuals with long COVID. Policymakers can use these insights to develop comprehensive strategies that prioritize public health, such as vaccination campaigns, vaccine mandates, and appropriate support for those affected by long COVID. Recognizing the employment disparities associated with long COVID and implementing supportive measures can contribute to a more inclusive and resilient labor market recovery for the benefit of all.

## 2. Materials and Methods

### 2.1. Survey Collection

The UHERO Rapid Health Survey is a cohort study that followed 2000 adult residents of Hawaii over time. Participants in the baseline cohort in May 2022 were asked to complete a follow-up questionnaire in November 2022. Out of the 2000 adults invited to participate in the follow-up, 1627 completed the survey, resulting in a retention rate of over 80%. The summary statistics section presents unweighted descriptive information on this sample.

Respondents were asked in both surveys if and when they most recently tested positive for COVID-19. Those who had tested positive were then asked the following: People who contracted COVID-19 often experience lingering effects from COVID-19. Have you had any lingering long-term effects of COVID-19? This was followed by asking the severity of the symptoms from 1—very mild to 5—very severe, and the length of symptoms from 1 to 6 months, with more than 6 months coded as 7 for this analysis. Additionally, we asked if respondents were employed, unemployed, or retired. In the November survey, respondents were asked if they had lost their jobs, if they had been furloughed, or had their hours reduced since May.

For this analysis, we selected respondents who completed both the May and November surveys. From this group, we further narrowed our focus to individuals who tested positive for COVID-19 at some point. Within this group, we separately analyzed those who tested positive in May (a sample of 391 individuals) and those who tested positive in November (a sample of 739 individuals). To examine specific long COVID symptoms, we only included individuals who reported experiencing long COVID, which consisted of 114 respondents from the May sample and 213 respondents from the November sample.

### 2.2. Data Analysis

To analyze the impact of Long COVID on unemployment, we used a probit regression on the employment variables with a binary outcome (i.e., yes/no to being unemployed, losing a job since May, or being furloughed or hours reduced since May).

A probit regression is a statistical model used to analyze the relationship between a binary dependent variable and a set of independent variables. The estimated coefficient can be used to estimate the change in the probability of the dependent variable being in one category relative to the reference category for a one-unit change in the corresponding independent variable, holding other variables constant (probit marginal effects) [21].

The model was used to estimate the impacts of having long COVID and demographic variables on employment outcomes among those who tested positive for COVID-19:

(1) $$EmploymentOutcome_{i}=\beta_{0}+\beta_{1}LongCOVID_{i}+\beta_{2}NumberCOVIDshots_{i}+X_{i}+e_{i}$$

where $EmploymentOutcome_{i}$ represents one of the three binary outcomes evaluated (being unemployed, losing a job since May, or being furloughed or hours reduced since May). $LongCOVID_{i}$ is equal to one if respondent *i* reported having lingering COVID-19 symptoms following infection. $NumberCOVIDshots_{i}$ is the number of COVID-19 vaccinations an individual has received where unvaccinated is coded as 0, only the initial vaccination as 1, with an additional 1 added for each booster shot. $X_{i}$ is a vector of demographic variables, including age (in years), gender, race, and highest education achieved. Race is categorized into African American, Caucasian, Chinese, Filipino, Hawaiian, Japanese, Latino, Samoan, Portuguese, and Other. The Caucasian dummy variable is omitted from the regressions to be used as the benchmark for comparison. Education achievement status is classified as less than high school diploma, high school diploma or equivalent, and college degree or higher. ‘Less then high school’ is omitted as the reference dummy variable. Lastly, $e_{i}$ represents the error term.

## 3. Results

As these results focus on the impacts of long COVID, all analyses only evaluate respondents who contracted COVID-19 at least once. All regression results report the probit marginal effects on the employment outcomes, with robust standard errors reported.

### 3.1. Summary Statistics

Table 1 reports the summary statistics for this dataset. Unemployment in this analysis includes both those unemployed and looking for work and not looking for work to capture anyone who left the labor market. Retired individuals are excluded from the analysis. Unemployment was slightly higher in November compared to May at 10.1% compared to the spring rate of 9.08%. Moreover, 5.35% of respondents lost their jobs between May and November, and 9.39% were either furloughed or had their hours reduced. Among those who reported having had COVID-19 in May (391 individuals), 29.2% reported having lingering symptoms (long COVID). While many more respondents reported contracting COVID-19 in November (739 individuals), the long COVID rate remained fairly stable at 28.8%.

The average length of these symptoms was higher in November by 0.862 months compared to May, which was expected, as this allowed for more time for symptoms to persist. The average severity of symptoms was also higher in November (2.469 out of 5), compared to May (2.377 out of 5). The average age of respondents was 48.92 years old. Our sample had a higher proportion of females than males, with 66% identifying as female. The most prominent racial and ethnic groups reported were Caucasian (27.8%), Japanese (20.3%), Hawaiian (18.1%), Filipino (11%), and Chinese (7.04%). The remaining ethnicities reported under 3% each.

### 3.2. Unemployment Results

The results of the regression for unemployment in May (columns 1–2) and November (column 3) are seen in Table 2. Reporting long COVID in May was correlated with a 6.4% higher likelihood of being unemployed and a 7.1% higher likelihood in November. Both results were significant. Reporting long COVID in November was positively correlated with being unemployed in November but with a lower magnitude and no statistical significance. Those who received more vaccination shots and older individuals were less likely to be unemployed, with each additional shot reducing likelihood by 4–7% and every 10 years in age reducing the likelihood by 18–33%.

### 3.3. New Job Loss, Furloughed, or Hours Reduced Results

The regression results, examining reports of job loss (columns 1–2) and being furloughed or having hours reduced (columns 3–4) since May are shown in Table 3. While there were no significant relationships found between having long COVID, all coefficients remain positive, showing that those reporting long COVID in both May and November had a higher incidence of experiencing these negative effects at that time.

### 3.4. Impacts of Length and Severity of Symptoms

Table 4 shows the results of limiting the sample to only those with long COVID to see how the length and severity of their symptoms impacted employment. Severity was ranked from 1—very mild to 5—very severe. For each additional level of severity reported in May, individuals were 6.36% less likely to be employed in May and 5.75% less likely in November. Some individuals may still have been sick with lingering symptoms at the time of the May survey, which means that we did not catch the full length of their illness. This may have led to the negative and significant signs observed between the length of symptoms in May and May unemployment.

### 3.5. Correlations between Comorbidities and Long COVID Symptoms

Table 5 and Table 6 show the pairwise correlations between self-reported comorbidities and long COVID symptoms, respectively. In Table 5, column (1) reports the correlations between each comorbidity condition and unemployment in May, while column (2) reports the same in November. At both times, depression and other mental health conditions were positively and significantly correlated with being unemployed, while hypertension was negatively and significantly correlated. In May, alcohol or substance use disorder and other chronic conditions were positively correlated with unemployment, while in November, intravenous drug use and other chronic lung conditions were positively correlated.

Table 6 shows that in May, only the severity of infection was significantly and positively correlated with unemployment. In November, only loss of taste and smell were positively and significantly correlated with being unemployed, while having a cough or other symptoms was negatively and significantly correlated with unemployment.

## 4. Discussion

We found a significant association between unemployment and contracting long COVID in Hawaii. Those who reported long COVID in May 2022 were 6.43% more likely to be unemployed at the time of this May survey and 7.07% more likely in November 2022. Reporting more severe lingering symptoms increased the likelihood of being unemployed as well.

The results from papers asking similar questions found similar but slightly different conclusions, possibly due to definitions of “long COVID.” Our survey follows US Center for Disease Control standards and asked the following: “People who contracted COVID-19 often experience lingering effects from COVID-19. Have you had any lingering long-term effects of COVID-19?” This was followed by questions regarding the specific symptoms they experienced, and the length and severity of these symptoms, with a minimum of 1 month for the length. Westerlind et al. [20] defined long COVID as symptoms persisting for at least 12 weeks in their study on Swedish sick leave following COVID infections, while Perlis et al. [17] defined it as symptoms lasting over 2 months. The UK’s current National Institute for Health and Care Excellence guidelines define long COVID as cases that involve ongoing symptomatic COVID-19 for 4 to 12 weeks, and post-COVID-19 syndrome when symptoms persist beyond 12 weeks [4].

Notably, throughout the results, we can see that the number of COVID-19 vaccinations received by May had a significant impact on respondents’ employment outcomes. This impact may have come about in a few different ways. First, the counties of Honolulu and Maui implemented vaccine mandate programs in September 2021, requiring employees and customers in the food and beverage, entertainment, and fitness industries to provide proof of vaccination or recent negative COVID-19 test results [22]. This mandate helped increase the state’s vaccination rate, with 94% of adults receiving at least the initial dose of the vaccine [1]. Our sample showed a very similar rate of vaccination, so we controlled each additional shot to estimate the employment impacts of increased vaccination. Additionally, the mandate was found to decrease foot traffic at businesses in the 14 weeks following its implementation; however, it also led to reduced rates of COVID-19 positivity and higher vaccination uptake during this time [23]. This study found that by the end of 2021, business foot traffic had returned to the level observed in non-mandate counties, and differences in positivity rates were diminished with the onset of the Omicron wave.

Currently, no studies have shown the direct impact of vaccination on employment; our study is the first to address this gap directly via a survey. Limbu et al. [24]’s meta-analysis found that unemployed individuals were more likely to be vaccine-hesitant compared to those employed across studies. As the state of Hawaii has a large population of Native Hawaiians, other Pacific Islanders, and Asians, the vaccination choices of these groups are important in understanding the impacts of the pandemic in the state. Prior to the vaccination or test mandates in the state, Native Hawaiians and other Pacific Islanders were less likely to be vaccinated, which was associated with higher infectivity rates among these populations [25]. Juarez et al. [26] found that among surveyed adults in Hawaii, Native Hawaiians and other Pacific Islanders were the only racial/ethnic groups to be more likely to trust and consume unofficial sources of COVID-19 information compared to Whites, which they found to be negatively correlated with the COVID-19 vaccination. This initial finding was extended by finding that similar measures of trust and consumption of unofficial information on COVID-19 were associated with reduced rates of booster uptakes as well [27]. Deng et al. [28]’s evaluation of COVID-19 vaccine booster hesitancy in Zhejiang Province, China, found that various factors led to vaccine hesitancy, including higher hesitancy among those younger and less educated. As we saw, older individuals were less likely to be unemployed in this study; this could have been tied to their increased likelihood of being vaccinated.

There were no significant differences observed in terms of sex or across different races, although this may have been due to the small sample size. However, we did observe a negative and significant correlation between age and unemployment. This could be because older adults tend to either be employed or retired, and retired individuals were not included in our sample. Additionally, we defined unemployed individuals as both those actively looking for work and those not currently in the labor market, which could have included young parents or students who were not actively seeking employment, regardless of their COVID-19 status or long COVID status. Nevertheless, we believed it was important to include these individuals in our analysis to capture those who were unable to look for work due to their long COVID symptoms as well as those who were discouraged from looking due to the current state of the economy.

We note that long COVID policies have attempted to address the challenges faced by individuals with persistent symptoms, but the condition’s impact on unemployment rates remains a significant concern. Continued efforts are needed to provide comprehensive support, accommodation, and reintegration opportunities for individuals with long COVID, mitigating the economic consequences and ensuring inclusive recovery.

Some limitations of this study are due to the nature of the survey data. There was potential for selection bias in the convenient sample and we did not adjust based on the state population. Younger individuals and men were underrepresented in our sample; however, based on our sample, we found that all major racial groups in the state, including Native Hawaiian, Asian, and White populations were represented. We also note that we found a higher unemployment rate among our respondents than the state’s reported levels, which may have been due to self-selection based on a higher valuation of an incentive payment or more time to complete the survey. Regardless, our survey included a wide range of individuals of diverse backgrounds and socioeconomic statuses across all of Hawaii. Additionally, given the unique economy and geographic location of the state of Hawaii, the results from this study may not be applicable to other regions or contexts. In particular, as a large sector of Hawaii’s economy includes jobs that are difficult to complete when one is ill (such as customer service or maintenance jobs within the tourism industry), different findings may occur in places with more opportunities to work from home or less physically demanding jobs.

## 5. Conclusions

We provide the first evidence on the link between long COVID and unemployment in Hawaii, which has significant implications for the economy, public health, the advancement of equality in business, and academia.

From an economic standpoint, our findings reveal a detrimental impact of long COVID on employment rates, exerting significant influence on the overall economy. Understanding the unique challenges faced by individuals with long COVID is essential for devising effective economic recovery strategies. Policymakers can utilize this knowledge to implement targeted support measures that address the labor market effects of the pandemic. Moreover, the negative economic consequences and associated costs identified in our research can serve as justifications for funding prevention programs, providing free vaccinations, or establishing vaccination requirements for specific groups. By recognizing the employment disparities associated with long COVID, we can work towards building a more inclusive and resilient economy that caters to the needs of all individuals.

From a public health perspective, which is a core focus of the *International Journal of Environmental Research and Public Health*, our study underscores the importance of considering the long-term health consequences of COVID-19. We observed an increased likelihood of unemployment among individuals with long COVID, emphasizing the need for comprehensive healthcare provisions, timely medical interventions, rehabilitation services, and mental health support. This understanding can inform public health policies aimed at preventing long COVID, effectively managing its impact, and promoting overall well-being in individuals affected by the condition.

Furthermore, our research has significant implications for advancing equality in business. By highlighting the employment disparities associated with long COVID, we highlight the importance of creating inclusive work environments that accommodate individuals with long-term health conditions, including long COVID. The findings underscore the critical importance of implementing policies that prioritize equitable access to healthcare, employment opportunities, and social support for all individuals.

Lastly, our study makes a valuable contribution to the academic literature by being among the first papers to examine the social and economic implications of long COVID, with a specific focus on employment. Our findings provide a foundation for future studies that will aim to quantify the costs associated with long COVID. Recognizing the economic burden imposed by this condition is crucial for policymakers, healthcare professionals, and researchers to develop effective strategies and allocate resources efficiently.

In conclusion, our study not only establishes the link between long COVID and unemployment in Hawaii but also highlights its profound implications for economics, public health, equality in business, and academia. By leveraging these findings, policymakers, healthcare professionals, and scholars can collaborate to develop targeted strategies, support systems, and policies that address the challenges faced by individuals with long COVID, promote economic recovery, and improve public health.

## Figures and Tables

**Table 1:** Summary of statistics.

	Observation	Mean	St. Dev.
Variables	(1)	(2)	(3)
Dummy Variables			
Unemployed in May	749	0.0908	0.287
Unemployed in November	745	0.101	0.301
Lost job since May	767	0.0535	0.225
Furloughed or reduced hours since May	767	0.0939	0.292
Reported long COVID in May	391	0.292	0.455
Reported long COVID in November	739	0.288	0.453
Highest education—Less than High School	765	0.379	0.485
Highest education—High School	765	0.0183	0.134
Highest education—College	765	0.6	0.49
Female	767	0.66	0.474
African American	767	0.00652	0.0805
Caucasian	767	0.278	0.448
Chinese	767	0.0704	0.256
Filipino	767	0.11	0.312
Hawaiian	767	0.181	0.385
Japanese	767	0.203	0.403
Korean	767	0.0169	0.129
Latino	767	0.0248	0.156
Samoan	767	0.00913	0.0952
Portuguese	767	0.0156	0.124
Other	767	0.0261	0.159
Discrete Variables			
Length of lingering symptoms in May (months)	114	3.57	2.238
Severity of lingering symptoms in May (1–5)	114	2.377	0.944
Length of lingering symptoms in November (months)	213	4.432	2.183
Severity of lingering symptoms in November (1–5)	213	2.469	0.944
Number COVID shots—May	758	1.951	0.799
Number COVID shots—November	758	2.509	1.097
Age (years)	766	48.92	15.4

**Table 2:** Unemployment results—probit marginal effects.

	Unemployed in May	Unemployed in November	
Variables	(1)	(2)	(3)
May—long COVID	0.0643 **	0.0707 **	
	(0.0299)	(0.0310)	
November—long COVID			0.0340
			(0.0232)
Number COVID shots—May	−0.0694 ***	−0.0395 **	
	(0.0161)	(0.0181)	
Number COVID shots—November			−0.00234
			(0.0108)
Age (years)	−0.00177 *	−0.00295 **	−0.00327 ***
	(0.00107)	(0.00115)	(0.000766)
Female	−0.0210	−0.0215	−0.00297
	(0.0328)	(0.0350)	(0.0232)
Highest education—high school	−0.0278	−0.118	0.0306
	(0.0690)	(0.0730)	(0.0864)
Highest education—college	−0.0808	−0.170 **	−0.00540
	(0.0692)	(0.0720)	(0.0855)
African American	0.0373	0.0259	0.0394
	(0.0982)	(0.106)	(0.103)
Chinese			−0.0521
			(0.0562)
Filipino	−0.0510	−0.0462	−0.0134
	(0.0521)	(0.0507)	(0.0385)
Hawaiian	0.0451	−0.0114	0.0327
	(0.0376)	(0.0417)	(0.0284)
Japanese	−0.00134	−0.0143	−0.0128
	(0.0437)	(0.0471)	(0.0320)
Latino	0.0811	0.0520	0.0163
	(0.0699)	(0.0739)	(0.0637)
Portuguese		0.0650	−0.0247
		(0.112)	(0.0870)
Other		0.0195	0.0605
		(0.0982)	(0.0600)
Observations	335	349	697
Pseudo R2	0.199	0.145	0.145

*** *p* < 0.01, ** *p* < 0.05, * *p* < 0.1, robust standard errors in parentheses.

**Table 3:** Change in employment between May and November results—probit marginal effects.

	Lost Job between May and November		Furloughed or Hours Cut bw May and November	
Variables	(1)	(2)	(3)	(4)
May—long COVID	0.0373		0.0164	
	(0.0241)		(0.0333)	
November—long COVID		0.0267		0.0358
		(0.0180)		(0.0247)
Number COVID shots—May	−0.0237 *		0.0291	
	(0.0141)		(0.0187)	
Number COVID shots—November		−0.00733		0.0164
		(0.00873)		(0.0118)
Age	−0.00163 **	−0.00191 ***	−0.00330 ***	−0.00255 ***
	(0.000816)	(0.000615)	(0.00105)	(0.000826)
Female	−0.0281	−0.0157	0.0114	0.0110
	(0.0239)	(0.0172)	(0.0322)	(0.0243)
Highest education—High School	0.456 ***	0.419 ***	−0.00602	−0.130 *
	(0.0916)	(0.0593)	(0.0798)	(0.0790)
Highest education—college	0.401 ***	0.383 ***	−0.0964	−0.185 **
	(0.0868)	(0.0564)	(0.0810)	(0.0783)
Chinese	0.0213	0.0282	−0.0522	−0.0167
	(0.0470)	(0.0347)	(0.0774)	(0.0484)
Filipino	−0.00432	0.00598	−0.148 **	−0.121 **
	(0.0332)	(0.0279)	(0.0599)	(0.0514)
Hawaiian	−0.0287	0.0141	−0.0499	0.00671
	(0.0312)	(0.0239)	(0.0408)	(0.0320)
Japanese	−0.0726 *	−0.0301	−0.0405	−0.0143
	(0.0415)	(0.0273)	(0.0423)	(0.0308)
Samoan		0.0593		0.0491
		(0.0715)		(0.112)
Portuguese	0.0728	0.0175		
	(0.0660)	(0.0584)		
Other	0.0832	0.0505	0.142 *	0.0777
	(0.0515)	(0.0445)	(0.0788)	(0.0602)
Observations	361	696	356	684
Pseudo R2	0.174	0.0929	0.124	0.0694

*** *p* < 0.01, ** *p* < 0.05, * *p* < 0.1, Standard errors in parentheses.

**Table 4:** Severity and length of symptoms on unemployment—probit marginal effects.

Variables	Unemployed in May	Unemployed in November	
Length of symptoms May (months)	−0.0364 **	−0.0164	
	(0.0180)	(0.0188)	
Severity of symptoms May (1–5)	0.0636 **	0.0575 *	
	(0.0314)	(0.0320)	
Length of symptoms Nov. (months)			−0.0129
			(0.00972)
Severity of symptoms Nov. (1–5)			0.0299
			(0.0256)
Number COVID shots—May	−0.138 ***	−0.0734 *	
	(0.0330)	(0.0395)	
Number COVID shots—November			0.00128
			(0.0218)
Age	−0.00410 **	−0.00450 **	−0.00303 **
	(0.00184)	(0.00213)	(0.00145)
Female	−0.117 **	−0.149 **	0.0220
	(0.0568)	(0.0672)	(0.0523)
Highest education—High School	−0.00215	−0.00559	0.747 ***
	(0.124)	(0.143)	(0.133)
Highest education—College	0.00837	−0.0719	0.712 ***
	(0.127)	(0.150)	(0.135)
Chinese			−0.0332
			(0.104)
Filipino	0.0299	−0.0341	0.00761
	(0.0850)	(0.0943)	(0.0742)
Hawaiian	−0.104	−0.211 **	−0.0186
	(0.0791)	(0.103)	(0.0632)
Japanese	0.00116	−0.109	−0.0178
	(0.0799)	(0.0918)	(0.0640)
Latino	0.203 **	0.152	0.0977
	(0.0883)	(0.0980)	(0.0914)
Portuguese		0.0918	0.0408
		(0.144)	(0.140)
Other		0.149	
		(0.160)	
Observations	99	104	200
Pseudo R2	0.377	0.284	0.0653

*** *p* < 0.01, ** *p* < 0.05, * *p* < 0.1, Standard errors in parentheses.

**Table 5:** Comorbidities and unemployment—pairwise correlations effects.

Comorbidities	May Unemployment	November Unemployment
Alcohol or substance use disorder	0.044 *	−0.002
	(0.081)	(0.927)
Asthma	0.007	−0.002
	(0.779)	(0.943)
Autoimmune disease	0.019	0.032
	(0.45)	(0.205)
Cancer diagnosis and/or treatment	0.003	−0.013
	(0.903)	(0.611)
Cardiovascular disease	−0.017	−0.035
	(0.509)	(0.16)
Chronic obstructive pulmonary disease	−0.04	−0.022
	(0.11)	(0.385)
Depression	0.130 ***	0.089 ***
	(0)	(0)
Diabetes	0.001	−0.014
	(0.977)	(0.57)
Hypertension	−0.099 ***	−0.096 ***
	(0)	(0)
Immunocompromised condition	0.036	0.026
	(0.153)	(0.292)
Intravenous drug use	0.039	0.048 *
	(0.123)	(0.053)
Other chronic lung condition	−0.033	0.049 **
	(0.194)	(0.048)
Prediabetes	−0.026	−0.031
	(0.306)	(0.219)
Other chronic conditions	0.061 **	0.018
	(0.015)	(0.462)
Other mental health conditions	0.046 *	0.093 ***
	(0.066)	(0)
Sickle cell anemia	−0.015	−0.014
	(0.542)	(0.571)
Observations	1599	1593

*** *p* < 0.01, ** *p* < 0.05, * *p* < 0.1.

**Table 6:** Long COVID symptoms and unemployment—pairwise correlations effects.

Long COVID Symptoms	May Unemployment	November Unemployment
Length of lingering symptoms (months)	−0.05	−0.039
	(0.597)	(0.578)
Severity (1—very mild to 5—very severe)	0.194 **	0.094
	(0.04)	(0.175)
Cough	0.058	−0.113 *
	(0.541)	(0.104)
Fatigue	−0.125	0.056
	(0.187)	(0.421)
Joint pain	0.019	0.04
	(0.845)	(0.566)
Loss of taste or smell	0.027	0.201 ***
	(0.772)	(0.003)
Mental Fog	0.117	0.034
	(0.215)	(0.629)
Other symptoms	−0.006	−0.161 **
	(0.947)	(0.02)
Observations	209	113

*** *p* < 0.01, ** *p* < 0.05, * *p* < 0.1.

## Data Availability

All data used for this project will be available de-identified as approved by the University of Hawaii Institutional Review Board upon request to the corresponding author.
